# Acceleration of slow autophagy flux induced by arabinofuranosyl cytidine improves its antileukemic effectiveness in M-NFS-60 cells

**DOI:** 10.55730/1300-0152.2619

**Published:** 2022-04-01

**Authors:** Salwa A. FOUAD, Gamal H. EL-SOKKARY, Abo Bakr ABDEL SHAKOR

**Affiliations:** Laboratory of molecular cell biology, Department of Zoology, Faculty of Science, Assiut University, Assiut, Egypt

**Keywords:** Arabinofuranosyl cytidine, hyperthermia, autophagy, myelogenous leukemia, M-NFS-60 cells

## Abstract

Arabinofuranosyl cytidine (AraC) is an analog of deoxycytidine used as an anticancer drug for leukemic patients. The effective dose always produces severe complications. The present study investigated the modulation of autophagy and its impact on the cytotoxicity of AraC toward murine myelogenous leukemia cells (M-NFS-60). Autophagy was inhibited by NH_4_Cl or Bafilomycin A1 or enhanced by amino acid starvation, glucose starvation, mild hyperthermia (41 °C), or rapamycin (Rap). Cells were treated with different concentrations, 0 to 2 μM, of AraC in the presence or absence of autophagy modulators. AraC-induced apoptosis is combined with autophagy, especially at lower concentrations. This autophagy is characterized by a slow flux, as indicated by levels of LC3B II and P62 proteins. Inhibition of autophagy did not alter cleaved caspase 3 levels (c-casp.3) or cell viability measured by MTT assays. Conversely, acceleration of AraC-induced autophagy by co-treatment with autophagy inducers reduced cell viability and increased c-casp.3 and c-PARP levels. Further, c-PARP levels were reduced in the presence of caspase inhibitor, Z-VAD-FMK. Enhancement of slow autophagic flux induced by low concentrations of AraC significantly increased the cytotoxicity of AraC toward M-NFS-60 cells. Such coadministration of autophagy inducers might improve the efficacy of AraC treatment and reduce effective doses.

## 1. Introduction

Arabinofuranosyl cytidine (AraC) (cytosine arabinoside or cytarabine) is a chemotherapeutic drug used alone or in combination with other anti-tumor agents to treat leukemias. AraC is an analog of deoxycytidine (dC) and became the most important drug for chemotherapy of acute myeloid leukemia more than three decades ago. Structural similarity between AraC and dC allows the former to be metabolized in the same metabolic pathways as dC. AraC is transported into cells in its nucleoside form and is then converted in a series of steps into the triphosphate (AraC-TP). AraC-TP competes with natural nucleoside triphosphate, dCTP, in proliferating cells for incorporation into DNA. This incorporation causes termination of daughter strand DNA synthesis and consequently kills leukemia cells (Perry, 2008). The low permeability of AraC across cell membranes dictates the use of high doses to achieve satisfactory antitumor activity (Novotny and Rauko, 2009). Unfortunately, such high-dose treatment is associated with toxic side effects, including anemia, thrombocytopenia, leukopenia, gastrointestinal disturbances, cerebellar toxicity, and fatality (Reese and Schiller, 2013). AraC has thus been combined with other agents to potentiate its anticancer effect and reduce adverse side effects (Novotny and Rauko, 2009).

Hyperthermia is used as adjuvant therapy for cancer. Hyperthermia is often used in combination with radiation therapy, chemotherapy, or radiochemotherapy. More recently, it has been used along with gene and immunotherapy. The main advantage of hyperthermia is an increased sensitivity of cells to chemotherapeutic agents or ionizing radiation (Issels, 2008). Autophagy can be induced by hyperthermia in various cancer cells. Signs of autophagy include rapid conversion of LC3 I into LC3 II, a decrease in p62/SQSTM1, and an increase in autophagic vacuoles AVs (Zhao et al., 2009; Krmpot et al., 2010; Yang et al., 2010; Ba et al., 2017).

Macroautophagy, usually referred to as autophagy, refers to the process of self-digestion of cytoplasmic components, such as aggregated/misfolded proteins and damaged organelles (Pyo et al., 2013). Cytoplasmic components are engulfed within double-membrane vesicles called autophagosomes. Autophagosomes fuse with lysosomes forming autophagolysosomes that are responsible for the enzymatic degradation of engulfed materials (Jin and Klionsky, 2014). Catabolic products of autophagy are released into the cytosol and recycled via bioenergetic pathways. Two principal functions of autophagy are to support the synthesis of new macromolecules and ATP and eliminate aggregated or misfolded proteins and damaged organelles. These functions allow cells to survive under metabolic stresses, such as nutritional depletion and hypoxia (Murrow and Debnath, 2013). Further, autophagy acts as a cellular defense mechanism against neurodegenerative diseases, genomic instabilities, and tumor initiation. The process also protects against infection by certain bacteria and viruses (Levine, 2005; Saha et al., 2018).

In oncology, simple connections between autophagy and cancer are difficult to identify because the role of autophagy is both complicated and controversial. The process can protect against malignant transformation and carcinogenesis by eliminating damaged organelles and recycling macromolecules (Mizushima and Klionsky, 2007). Autophagy seems to play either a prodeath or a prosurvival role that may increase or diminish the efficacy of chemotherapy. Autophagy can promote cell death either by enhancing apoptosis or modulating autophagic cell death (Sui et al., 2013). Several studies report that autophagy is involved in leukemia cell death induced by antineoplastic agents (Puissant et al., 2010; Calabretta and Salomoni, 2011; Han et al., 2011; Schnekenburger et al., 2011; Cheng et al., 2012; Mahoney et al., 2012).

We investigated the role of autophagy in the modulation of leukemia cell death induced by AraC. Further, we examined whether this modulation increases the effectiveness of AraC. The objective was to supply information for future improvement of the therapeutic efficacy of AraC for the treatment of leukemia.

## 2. Materials and methods

### 2.1. Chemicals and reagents

RPMI medium with L-glutamine, fetal bovine serum (FBS), and antibiotic mix (10,000 units penicillin/mL and 10,000 units streptomycin/mL) were purchased from Gibco (Invitrogen, CA. USA). RPMI-1640 medium with L-glutamine and without D-glucose (-Glu) was obtained from Biochrome Ltd (Cambridge, UK). RPMI 1640 medium without amino acids (-AA) was acquired from US biological life science. Arabinofuranosyl cytidine (AraC), carbobenzoxy-valyl-alanyl-aspartyl-[O-methyl]-fluoromethylketone (Z-VAD-FMK), bafilomycin A1, monodansyl cadaverine (MDC), rapamycin, holotransferrin, and rabbit polyclonal anti-P62/SQSTM1 IgG antibody were purchased from Sigma Aldrich. Enhanced chemiluminescent (ECL) substrate was obtained from (Thermo Scientific, Rockford, USA). Rabbit polyclonal anti-PARP IgG antibody was purchased from Invetrogen. Rabbit polyclonal anti-cleaved caspase-3 IgG and rabbit anti-β actin IgG antibodies were from ABCAM. Rabbit anti-LC3B IgG antibody was from Cell Signaling Technology. Horseradish peroxidase (HRP) conjugated goat anti-rabbit IgG was obtained from Santa Cruz Biotechnology. Other chemicals were purchased from trusted local suppliers.

### 2.2. Cell culture

Murine myelogenous leukemia M-NFS-60 cells were purchased from VACSERA –Cell Culture Unit (Dokky, Giza, Egypt). All experiments were performed using mycoplasma-free cells. Cells were cultured in RPMI-1640 medium with L-glutamine supplemented with 10% (v/v) heat-inactivated FBS, 100 units/mL penicillin, and 100 μg/ mL streptomycin at 37 °C in a humidified atmosphere of 95% air and 5% CO_2_. Cells were cultured in D-glucose or amino acid-deficient RPMI medium for 3 h before drug treatment to induce autophagy.

### 2.3. Autophagy modulation

RPMI without D-glucose was supplemented with 1 μg/ mL transferrin and insulin for glucose starvation medium (-Glu). For amino acid starvation medium (-AA), RPMI without amino acids and without D-glucose was supplemented with 1 μg/mL transferrin and insulin and 20 mM D-glucose. Cells were washed twice with PBS and incubated in starvation medium for 3 h with or without AraC treatment. M-NFS-60 cells were incubated in a controlled water bath at 41 °C for 60 min for hyperthermia-induced autophagy experiments. Cells were then returned to a controlled 37 °C incubator with or without AraC for 24 h. The temperature was monitored with a reference thermometer secured in a plate-like the one used for cells. For induction of autophagy by rapamycin, cells were cultured in 200 ng/mL rapamycin for 3 h with or without the AraC. Finally, autophagy was inhibited by incubation of cells with 5 mM NH_4_Cl or 0.5 μM Baf-A1 for 1 h before incubation with AraC. Both NH_4_Cl and Baf-A1 were retained in the growth medium during subsequent incubation.

### 2.4. MDC staining

Autophagic vacuoles were labeled by incubating cells with 0.05 mM MDC in PBS at 37 °C for 10 min. Cells were then washed three times with PBS. Stained cells were mounted on glass slides and examined using a fluorescence microscope (Nikon, Germany). Images from different fields of the same slide for each treatment were obtained. Total MDC puncta were counted in 300 cells for each treatment, and the average number of puncta per 100 cells was calculated.

### 2.5. MTT assay

Cells (2 × 10^3^ cells/well in 96-well plates) were incubated with AraC alone or in combination with autophagy inhibitors or inducers. The medium was replaced with 200 μL of fresh medium containing 2 mg/mL MTT per well and incubated for 3 h at 37 °C. Formazan crystals were dissolved in 200 μL of DMSO and quantified with absorbance at 570 nm and a reference wavelength of 630 nm. Data were expressed as the average percentage of viable cells compared to controls.

### 2.6. Acridine orange/ethidium bromide (AO/EB) staining

Ethidium bromide and acridine orange are intercalating, nucleic acid-specific fluorochromes which emit green and orange fluorescence, respectively. Viable cells display bright green nuclei with intact structures. Apoptotic cells show orange nuclei with condensed chromatin and necrotic cells display orange nuclei with intact structure (Kasibhatla et al., 2006). Cell suspensions (0.5 × 10^6^ cells/ mL) were centrifuged at 1500 rpm for 5 min at room temperature and pellets were washed with 300 μL PBS. Pellets were then resuspended in 100 μL of PBS. Five μL of AO/EB dual dye stock solution containing 100 μg/ mL of each dye was added and cells were incubated for 5 min. Twenty-five μL of cell suspension was placed onto a microscopic slide and immediately examined with a fluorescence microscope.

### 2.7. Western blotting analysis

Cells were centrifuged at 1500 rpm for 5 min at room temperature, pellets washed twice with ice-cold PBS, and cells lysed with ice-cold RIPA buffer (50 mM Tris-Cl [pH 7.6], 5 mM EDTA, 150 mM NaCl, 0.5% NP-40, and 0.5% Triton-X-100) containing 1 μg/mL leupeptin and aprotinin, and 0.5 mM PMSF. Lysates were centrifuged at 2500 rpm for 10 min at 4 °C. Protein concentrations were measured by Bradford assay. Forty μg protein aliquots were separated by SDS–PAGE using 10% gels (12% for LC3) and then transferred to nitrocellulose membranes. Membranes were blocked with 2% BSA and probed with primary antibodies (anti-c-casp.3, anti-p62/SQSTM1, anti-PARP, anti-LC3B, and anti-β-actin, 1:1000) overnight at 4 °C. Membranes were then incubated with horseradish peroxidase-conjugated secondary antibody (1:10,000) for 1 h at room temperature. Detection was performed using the ECL substrate.

### 2.8. Statistical analysis

Quantitative results are expressed as means ± SD of three independent experiments. Statistical significance of differences was analyzed with one-way analysis of variance followed by Newman-Keuls post-test. Results were considered statistically significant at p < 0.05.

## 3. Results

### 3.1. Induction of apoptosis and autophagy by AraC

**Figure 1:**
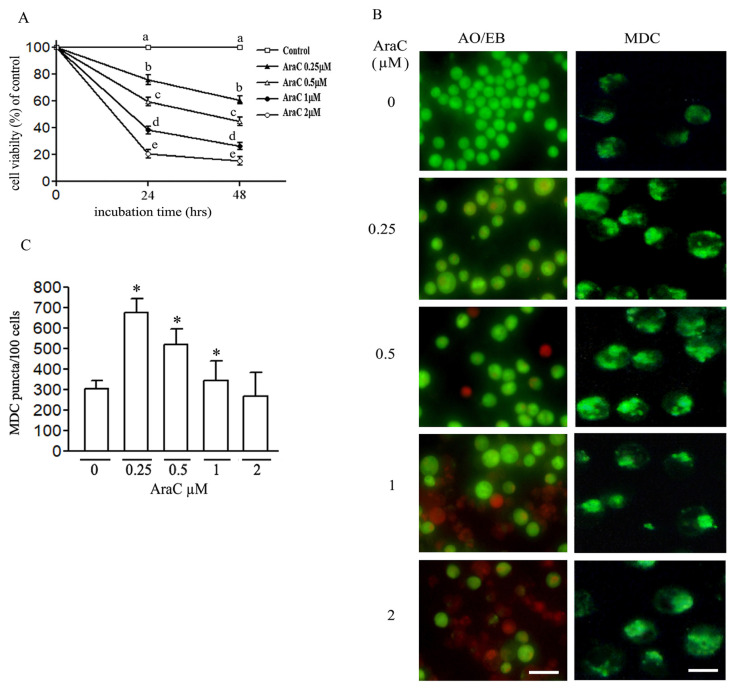
Induction of apoptosis and autophagy by different concentrations of AraC. (A) MTT assay after 24 and 48 h. Values at the same time with unlike superscript letters are significantly different (p < 0.05). (B) Autophagy and apoptosis were detected by MDC and AO/EB staining, respectively, Bar = 5μm. (C) MDC puncta were quantified from three independent experiments, n = 300 cells, (* or # p < 0.05).

The toxicity of AraC toward M-NFS-60 cells was examined after incubation for 24 and 48 h across a range of concentrations −0, 0.25, 0.5, 1, and 2 μM. Cell viability was assessed with MTT assays (Figure 1A). The reduction of cell viability was time- and dose-dependent. Cell death was also examined morphologically by AO/EB double staining, which showed that the percentage of apoptotic cells (pre and late stages) with orange to red nuclei and with different degrees of chromatin damage increased in a dose-dependent manner (Figure 1B). The presence of necrotic cells was not evident across AraC concentrations.

Induction of autophagy by AraC in M-NFS-60 cells was examined by MDC, a specific autophagy stain (Figure 1B). The formation of autophagosomes was visible at lower concentrations of AraC (0.25 and 0.5 μM) but not in higher concentrations (1 and 2 μM). MDC puncta per 100 cells increased at lower AraC concentrations (Figure 1C).

### 3.2. Inhibition of AraC-induced autophagy did not affect cell death

**Figure 2:**
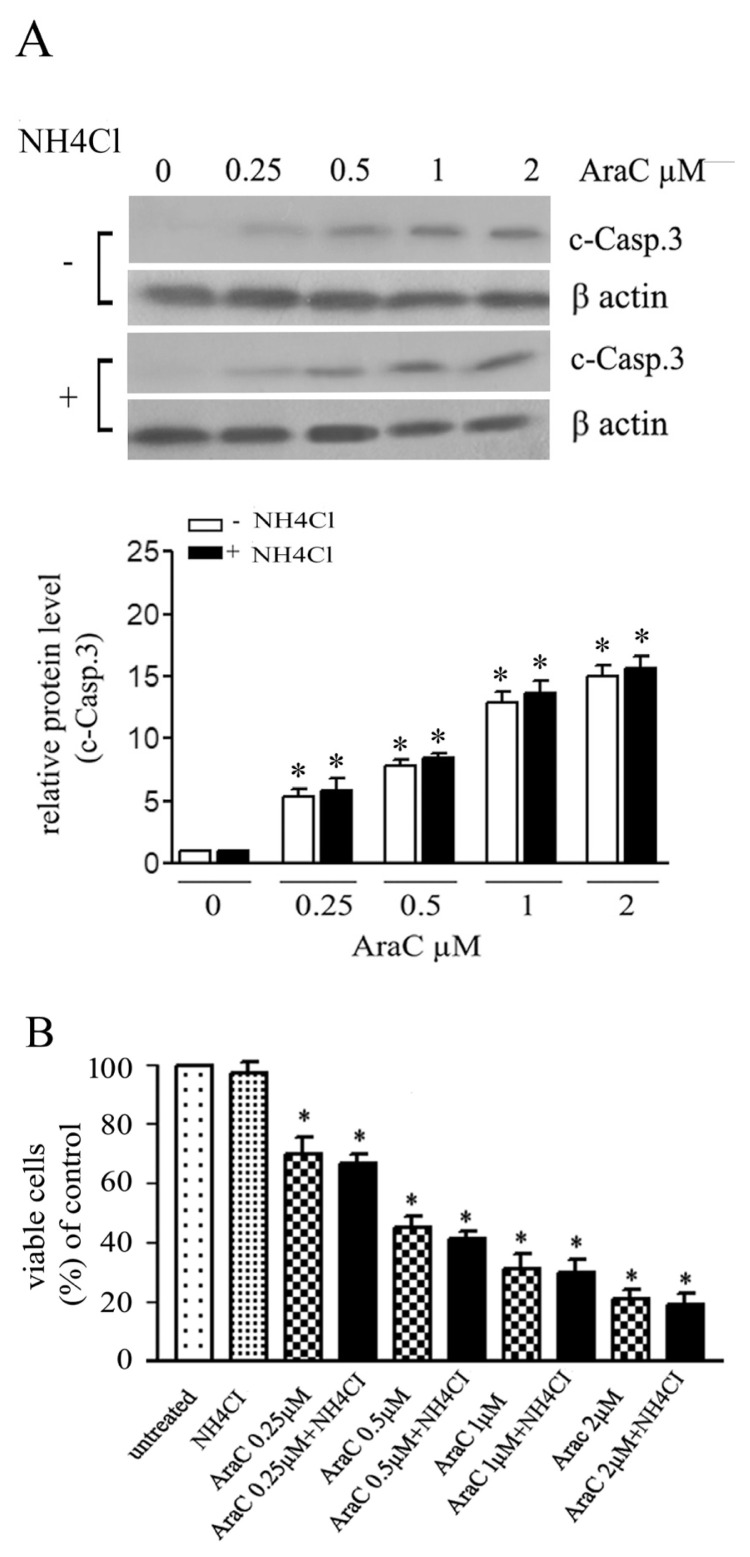
Autophagy inhibition shows no significant effects on apoptotic cell death induced by AraC. Cells were treated with different concentrations of AraC alone or combined with 5 mM NH_4_Cl for 24 h. (A) western blotting analysis of c-casp.3 levels. Relative protein levels were quantified from three independent experiments and presented in bar graphs. Densitometric data are means ± SD values (* p < 0.05). (B) MTT assay after 48 h. Data are means of three independent experiments ± SD (* p < 0.05).

Levels of c-casp.3 were analyzed by western blotting with or without autophagy inhibition by NH_4_Cl. c-casp.3 levels increased significantly after AraC treatment in a dose-related manner (Figure 2A). Inhibition of autophagy by NH_4_Cl had no significant effect on c-casp.3 levels compared NH_4_Cl untreated cells. Cell viability was examined after 24 h treatment at different concentrations of AraC with or without NH_4_Cl using MTT assays (Figure 2B). No significant difference in cell viability between AraC treated and AraC- and NH_4_Cl-co-treated cells were observed. Thus, inhibition of autophagy by a weak lysosomal tropic agent, NH_4_Cl, has no significant effect on cell death induced by AraC, especially at the lower concentrations when autophagy is prominent.

### 3.3. AraC-induced autophagy shows a slow flux

**Figure 3:**
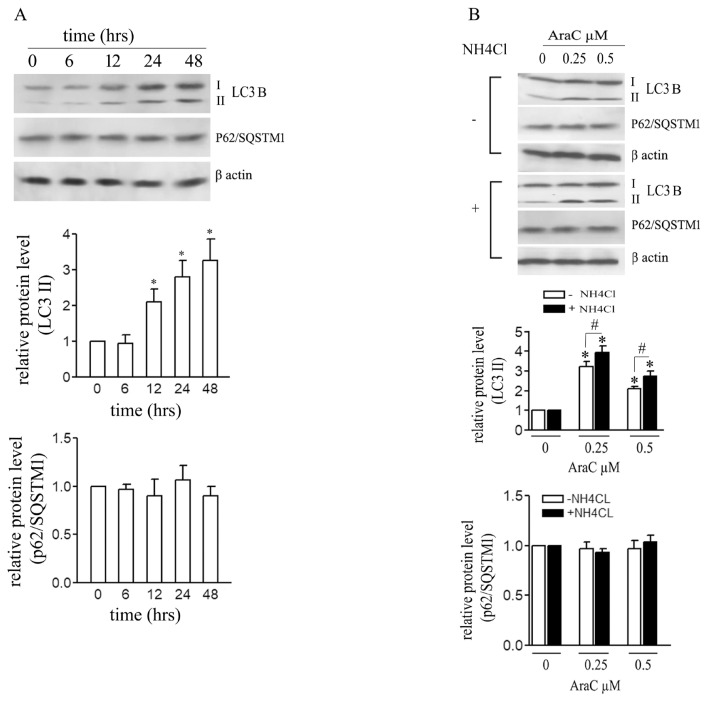
AraC induces slow autophagy flux. Cells were treated with AraC (0.5 μM) for 48 h. (A) LC3B II and P62/SQSTM1 levels at different time points were analyzed by western blotting. (B) LC3B II and P62/SQSTM1 levels were analyzed by western blotting after 24 h incubation with AraC (0.25 and 0.5 μM) with and without 5 mM NH_4_Cl. Relative protein levels were quantified densitometrically. Data are means of three independent experiments ± SD values (*or # p < 0.05).

The induction of autophagy by 0.5 μM AraC was examined by western blotting for levels of LC3B and P62/SQSTM1 at different times up to 48 h (Figure 3A). Expression of LC3B II increased significantly after 12 h then gradually increased further until 48 h. No significant difference in expression of p62/SQSTM1 between control and AraC treated cells was observed (Figure 3A). AraC-induced autophagy was next assessed by monitoring levels of LC3B II and P62/SQSTM1 with and without NH_4_Cl (Figure 3B). NH_4_Cl cotreatment increased LC3B II expression compared with cells treated only with AraC. The increase was small but significant. Again, P62/SQSTM1 expression did not change significantly. AraC thus induces slow autophagy flux, at least in the time frame of our experiment, 48 h.

**Figure 4:**
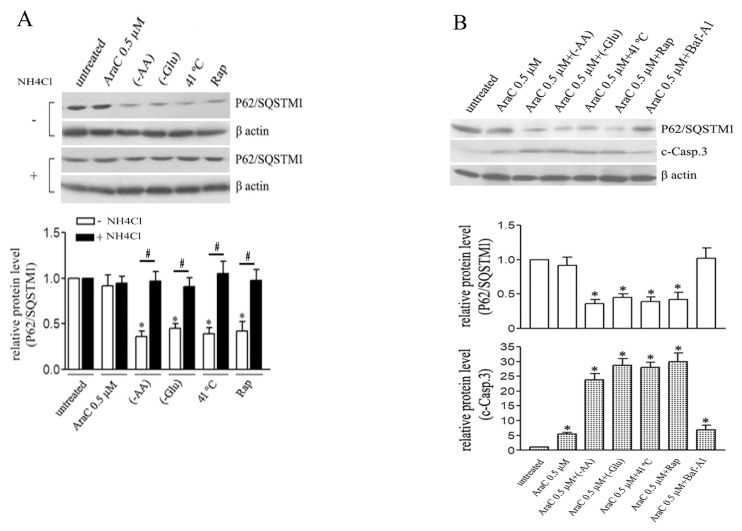
External autophagy inducers accelerate AraC-induced slow autophagy flux. Cells were treated with AraC (0.5 μM) for 48 h. (A) Western blotting analysis of the level of P62/SQSTM1 after treatment with AraC, amino acid starvation (-AA), glucose starvation (-Glu), mild hyperthermia (41 °C for 60 min) or Rapamycin (Rap) in the presence or absence of NH_4_Cl. (B) western blotting analysis of the level of P62/SQSTM1 after treatment with AraC alone or in combination with the mentioned autophagy inducers or inhibitor (Baf-A1) as shown. The relative protein levels were quantified densitometrically. Data are means of three independent experiments ± SD (*or # p < 0.05).

We also compared the expression of P62/SQSTM1 after 0.5 μM AraC treatment with and without different well-known autophagy inducers, including amino acid starvation (-AA), and glucose starvation (-Glu), mild hyperthermia (41 °C for 60 min) and rapamycin (Rap). These inducers all enhanced the degradation of P62/SQSTM1 (Figure 4A). Conversely, cotreatment with NH_4_Cl inhibited such degradation. Slow autophagy induced by lower AraC concentrations is related to its mechanism of action but not to the nature of autophagy itself in this cell line.

### 3.4. Acceleration of AraC-induced slow autophagy evokes apoptosis

Cells were treated with 0.5 μM AraC alone or combined with -AA, -Glu, 41 °C or Rap. First, levels of both c-casp.3 and P62/SQSTM1 were used to assess the simultaneous effects of autophagy modulation on apoptosis (Figure 4B). c-casp.3 expression was significantly elevated after cotreatment with AraC and autophagy inducers compared with cells treated with AraC alone. Conversely, cotreatment with autophagy inducers enhanced the degradation of P62/SQSTM1, and Baf-A1 pretreatment eliminated such degradation (Figure 4B). Inhibition of AraC-induced autophagy by Baf-A1 did not cause significant changes in c-casp.3 levels compared with cells treated with AraC alone.

**Figure 5:**
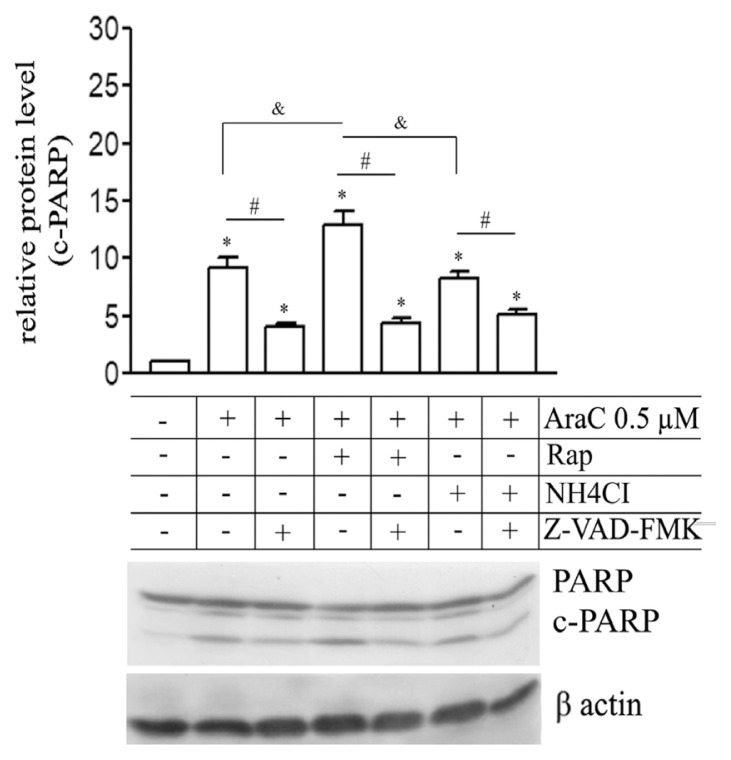
Autophagy induction but not inhibition augments apoptotic cell death induced by AraC. Western blotting analysis of c-PARP levels after AraC (0.5 μM) treatment for 48 h. alone or combined with Rapamycin (Rap) or NH_4_Cl in the presence or absence of Z-VAD-FMK; an inhibitor of caspases. Relative protein levels of c-PARP were quantified from three independent experiments and presented in bar graphs. Densitometry Data are means of three independent experiments ± SD *, # or & p < 0.05).

For further confirmation, the level of c-PARP was analyzed by western blotting with and without Z-VAD-FMK; an inhibitor of caspases (Figure 5). AraC (0.5 μM) significantly upregulated c-PARP compared with control cells; this effect was inhibited by Z-VAD-FMK. Levels of c-PARP sharply increased with AraC exposure and Rap cotreatment compared with AraC alone. This increase almost returns to its basal level in the presence of Z-VAD-FMK. The inhibition of autophagy by NH_4_Cl has no significant effect on c-PARP expression induced by AraC, but Z-VAD-FMK reduces c-PARP levels observed after AraC and NH_4_Cl cotreatment. Autophagy flux enhancement but not inhibition promotes apoptotic properties of AraC is thus apparent.

### 3.5. Acceleration of AraC-induced slow autophagy flux potentiates antileukemic effects

**Figure 6:**
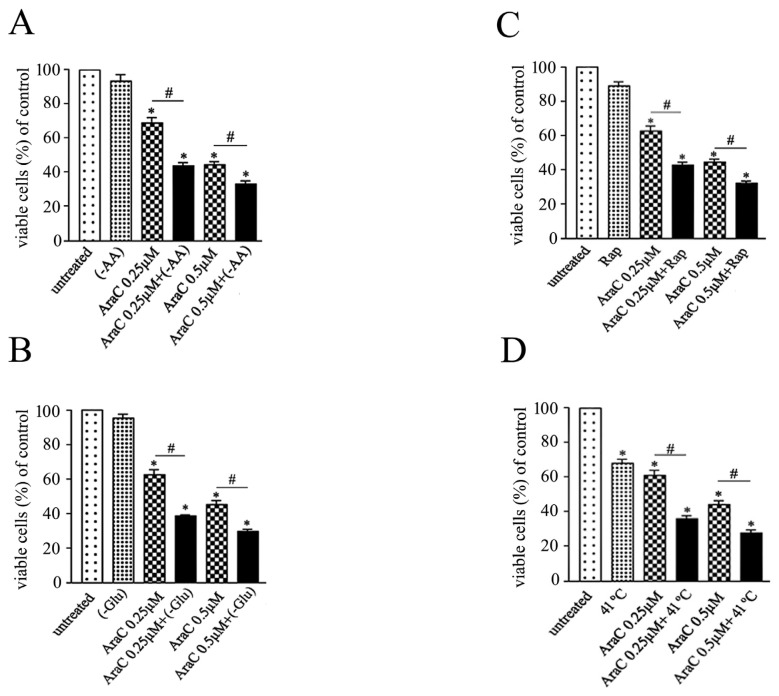
Autophagy induction promotes AraC antileukemia activity. Cell viability was determined by MTT assay after 48 h of treatment with AraC (0.25 and 0.5 μM) alone or in combination with (A) amino acid starvation (-AA), (B) glucose starvation (-Glu), (C) Rapamycin (Rap) or (D) mild hyperthermia (41 °C for 60 min). Data are means of three independent experiments ± SD (* or # p < 0.05).

Cell viability after 48 h exposure to AraC (0, 0.25, and 0.5 μM) alone or in combination with autophagy inducers indicates that -AA, -Glu, and Rap do not affect cell viability significantly (Figure 6A, B, and C). Mild hyperthermia (41 °C for 60 min) significantly reduces viability (Figure 6D). Cotreatment of AraC with -AA, -Glu, mild hyperthermia, or Rap significantly reduces viability compared with cells treated with AraC alone. Acceleration of the slow autophagy flux induced by AraC thus potentiates its activity against leukemia cells.

## 4. Discussion

Apoptosis caused by AraC is well established in a variety of leukemia cells in vitro (de Vries et al., 2006). In the present study, AraC significantly reduced, in a dose-dependent manner, the viability of M-NFS-60 myelogenous leukemia cells concurrent with induction of apoptosis. These findings are consistent with Bosnjak et al. (2014), who used human lymphoblastic leukemia cell line REH and Vincelette and Yun (2014), who used acute myelogenous leukemia and acute lymphocytic leukemia cell lines. Moreover, Chen et al. (2017) reported that treatment with increasing concentrations of AraC from 50 nmol/L to 50,000 nmol/L showed growth inhibition in a dose-dependent manner in eight different myeloid leukemia cell lines.

Autophagy induction by AraC in different leukemia cell lines and primary leukemia cells was previously reported by Bosnjak et al. (2014) Cheong et al. (2016), and Chen et al. (2017). Induction of autophagy by AraC in M-NFS-60 cells in the present study was demonstrated by the formation of autophagosomes stained with MDC and the expression of LC3B II. No significant changes in expression of p62/SQSTM1 were observed in either untreated cells or the presence of AraC. LC3B II expression increased slowly over 48 h of drug treatment. Autophagy inhibition produced no detectable change in P62/SQSTM1, but levels of LC3B II were slightly but significantly increased. Autophagy is thus induced but with a slow flux. AraC seems to initiate autophagy but degradation is slow or undetectable, at least in the 48-h time frame of the experiments. Perhaps this basal expression of p62/SQSTM1 and accumulation of autophagosomes are needed for AraC-mediated control of autophagy.

**Figure 7:**
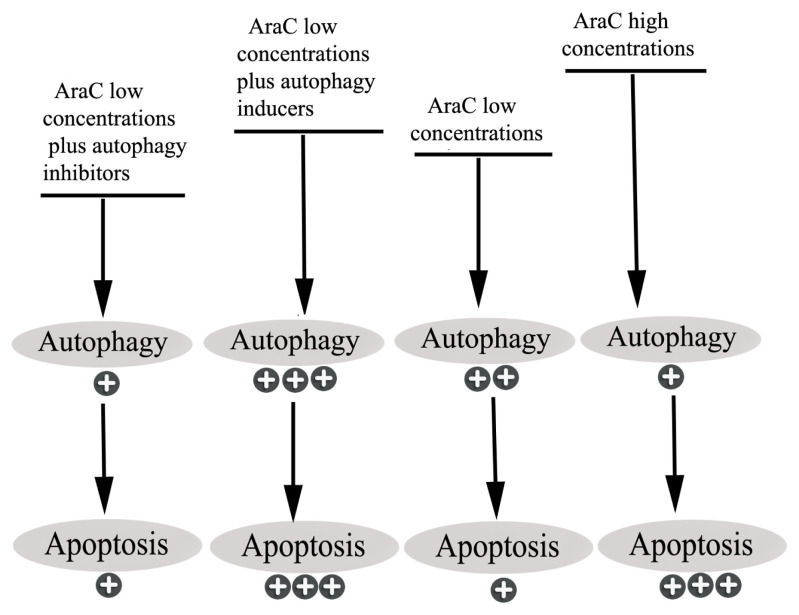
Schematic drawing showing the effect of high and low concentrations of AraC with autophagy inhibitors or inducers on autophagy and apoptosis.

The role of autophagy in regulating cancer cell death or survival is still controversial. Certain studies demonstrate that apoptotic cell death during chemotherapy is induced by autophagy (Fanzani et al., 2011; Notte et al., 2011), however, other studies suggest that autophagy prevents apoptosis in cancer cells (Liu et al., 2011; Zhao et al., 2014). The present study shows that autophagy inhibition by NH_4_Cl or Baf-A1 does not potentiate AraC antileukemic effects. Thus, autophagy induced by AraC shows no benefit toward cell survival. Inhibition of autophagy by 3MA or by knockdown of beclin-1 also did not affect AraC (0.05 μM) induced apoptosis in U937 cells (Chen et al., 2017). Conversely, inhibition of autophagy by Baf-A1, chloroquine, 3MA, or knockdown of LC3B or p62 markedly increased apoptosis in leukemia cells treated with AraC at concentrations of 3.2 or 10 μM (Bosnjak et al., 2014; Cheong et al., 2016). Accordingly, Palmeira-dos-Santos et al. (2014) hypothesized that the stage of autophagy inhibition is crucial for the responses of HL-60 leukemic cells to AraC. 3MA, which inhibits the initial stage of autophagy by blocking class III phosphoinositide 3-kinase-dependent formation of autophagosome, increased the antiproliferative activity of AraC (10 μM). Also, the authors reported no further effects when chloroquine, which inhibits the late stage of autophagy by blocking autolysosome acidification and autophagic proteolysis, was combined with AraC. This apparent contradiction might reflect the variety of AraC concentrations used by different researchers, variable exposure times, or cell line-specific responses. In the present study, low concentrations of AraC stimulated autophagy more prominently than higher concentrations. The results of high or low concentrations of AraC with autophagy inhibitors or inducers, based on the current study are summarized in (Figure 7).

Autophagy by -AA, -Glu, mild hyperthermia (41 °C for 60 min), or Rap combined with AraC resulted in increased toxicity and apoptosis compared with AraC alone. Thus, the enhancement of autophagy promotes apoptosis induced by AraC in leukemia cells. AraC-induced autophagy, therefore, appears to promote cell death. This drug (0.5 μM) or autophagy inducers alone induce little apoptosis, but cotreatment augments the overall effect. Autophagy inducers may accelerate autophagy induced by AraC, thus exceeding the threshold between autophagy and apoptosis.

The apoptotic effect of AraC is p53-dependent, since the FLT3-ITD leukemia cell line, which harbors mutant TP53, is AraC-resistant (Ko et al., 2019). Perhaps AraC-induced autophagy is also p53-dependent. AraC is a modified nucleotide, making DNA damage upon replication, this damage stimulates the fixation pathway of p53. Low doses of the drug may induce little DNA damage permitting P53 pathway and hence autophagy to start working. Whereas the effect of high doses is beyond the fixation capacity of p53 and accordingly P53 apoptotic pathway is stimulated. Both autophagy and P53 have mutual and complex regulatory machinery, and reciprocal control is highly dependent on the cell cycle (Tasdemir et al., 2008). The role of p53-dependent autophagy in apoptotic death of acute leukemia cells is further confirmed using inhibition of MDM2, an E3 ubiquitin ligase of p53 that promotes its proteasomal degradation, by Nutlin 3a (Borthakur et al., 2015). Such inhibition encourages p53-dependent autophagy and subsequent cell death. A suggested role for autophagy following AraC exposure is to supply the energy needed for P53 downstream signaling to repair AraC-induced genotoxic defects. This defect is, however, difficult to repair, and p53-dependent autophagy may instead accelerate cell death. Also, these processes may explain why low concentrations of the drug stimulated autophagy induction more efficiently than higher concentrations. Lower concentrations cause fewer genotoxic defects, thus allowing more time for the p53-autophagy system to repair the damage. Higher concentrations may overwhelm this repair system and lead to apoptosis.

## 5. Conclusion

AraC induces slow autophagy in (M-NFS-60) cells. Acceleration of slow autophagy enhances the apoptotic properties of the drug. Conversely, inhibition of autophagy did not affect apoptosis. We suggest that AraC-induced autophagy is not a prosurvival mechanism, and instead accounts for its antineoplastic effects. Accordingly, promoting AraC-induced autophagy by cotreatment with autophagy inducers might be useful for reducing effective drug doses and ameliorating adverse effects.
